# Comparing the clinical characteristics and outcomes of septic shock children with and without malignancies: a retrospective cohort study

**DOI:** 10.1016/j.jped.2024.06.003

**Published:** 2024-07-03

**Authors:** Haixin Huang, Ruichen Zhang, Jian Chen, Hongxing Dang, Chengjun Liu, Siwei Lu, Yue-qiang Fu

**Affiliations:** Children's Hospital of Chongqing Medical University, Department of Critical Care Medicine, National Clinical Research Center for Child Health and Disorders, Ministry of Education Key Laboratory of Child Development and Disorders, Chongqing Key Laboratory of Pediatrics Metabolism and Inflammatory Diseases, Chongqing, China

**Keywords:** Septic shock, Children, Malignancies, Mortality

## Abstract

**Objective:**

There is an amelioration in mortality rates of septic shock patients with malignancies over time, but it remains uncertain in children. Therefore, the authors endeavored to compare the clinical characteristics, treatment needs, and outcomes of septic shock children with or without malignancies.

**Methods:**

The authors retrospectively analyzed the data of children admitted to the PICU due to septic shock from January 2015 to December 2022 in a tertiary pediatric hospital. The main outcome was in-hospital mortality.

**Results:**

A total of 508 patients were enrolled. The proportion of Gram-negative bacteria and fungal infections in children with malignancies was significantly higher than those without malignancies. Septic shock children with malignancies had a longer length of stay (LOS) in the hospital (21 vs. 11 days, p<0.001). However, there were no statistically significant differences in the LOS of PICU (5 vs. 5 days, *p* = 0.591), in-hospital mortality (43.0 % vs. 49.4 %, *p* = 0.276), and 28-day mortality (49.2 % vs. 44.7 %, *p* = 0.452). The 28-day survival analysis (*p* = 0.314) also showed no significant differences.

**Conclusion:**

Although there are significant differences in the bacterial spectrum of infections, the septic shock children with or without malignancies showed a similar mortality rate. The septic shock children with malignancies had longer LOS of the hospital.

## Introduction

Sepsis is a life-threatening organ dysfunction caused by the host's dysfunctional response to infection,^1^ which is one of the main causes of hospitalization and death in children.^2^ Malignancies perennially loom as a high-risk factor for sepsis.^3^ Within a study^4^ encompassing exceeding one million hospitalized patients with sepsis, over one-fifth of patients were associated with cancer. Another study^5^ mentioned that two of the three most common potential causes of death in sepsis patients were related to malignancies (solid tumor cancer [21.0 %] and hematological diseases [10.3 %]). Sepsis patients with malignancies are also an important component of ICU patients (cancer [15.7 %] and hematologic cancer [2.9 %]).^6^ The risk of multiple organ failure in sepsis patients with malignancies is higher than those without malignancies,^7^ and the mortality rate of sepsis and septic shock patients with malignancies is quite high (69.4 %).^8^ While some studies^3^^,^^9^ have indicated an amelioration in mortality rates over time, it is imperative to underscore that the bulk of these studies predominantly pertain to adults. Furthermore, the morbidity of malignancies in children may be also different from those in adults.

The authors plan to conduct a study aimed at enhancing the insight into children affected by septic shock, particularly those with malignancies. The authors speculate that septic shock children with malignancies have a worse prognosis than those without malignancies. To verify the present hypothesis, the authors plan to compare the clinical characteristics, treatment needs, and outcomes of septic shock children with or without malignancies.

## Methods

### Study design

A retrospective cohort study was undertaken to analyze clinical data and prognostic outcomes in pediatric patients experiencing septic shock admitted to the Pediatric Intensive Care Unit (PICU) at Children's Hospital, Chongqing Medical University, during the period spanning January 1, 2015, to December 31, 2022. The Institutional Review Board of Children's Hospital, Chongqing Medical University, granted approval for this study. Due to the retrospective design, the requirement for informed consent was waived.

The attending physicians of the pediatric patients diagnosed severe infections by assessing their clinical presentation, laboratory findings, and imaging results. Septic shock was determined as a severe infection leading to cardiovascular dysfunction, including hypotension, insufficient fluid resuscitation, the requirement for vasoactive drugs, or perfusion damage, in accordance with the SCCM guidelines of 2005^10^ and the pediatric guidelines of 2020.^2^

Patients were identified using discharge diagnosis data from the electronic medical databases. The inclusion criteria were patients who were diagnosed with septic shock and admitted to the pediatric intensive care unit (PICU) due to septic shock. The exclusion criteria included: 1. Patients diagnosed with septic shock in other hospital departments but not transferred to PICU, 2. Patients with severe viral infection (such as severe adenovirus pneumonia), 3. Children concomitant with septic shock before abdominal surgery in the surgical ward who underwent surgery and transferred to PICU for postoperative ventilation support rather than anti-shock treatment, and subsequently transferred back to the surgical ward on the following day, 4. Patients with sepsis without septic shock.

### Data collection and definition of variables

Demographic and clinical data were collected, encompassing age, gender, comorbidities (tumors are divided into solid tumors and hematological tumors), and laboratory values such as white blood cell count (WBC), hemoglobin (Hb), platelet count (PLT), C-reactive protein (CRP), procalcitonin (PCT), lactate, bilirubin, creatinine, activated partial thromboplastin time (APTT), international normalized ratio (INR), D-dimer, fibrinogen, and culture results. Blood samples for PCT, CRP, and other blood parameters were collected at the time of admission.

The authors also obtained disease severity scores, including the International Society of Pediatric Index of Mortality (PIM)-3,^11^ and Pediatric Sequential Organ Failure Assessment (pSOFA) scores.^12^ Additionally, the authors documented the requirement for continued renal replacement therapy (CRRT) and mechanical ventilation (MV), along with the durations of CRRT and MV.

### Outcomes

The primary focus of this study was to evaluate the frequency of in-hospital mortality at any point during the patient's hospitalization. Secondary endpoints included the duration of stay in the PICU, the length of hospital stay, as well as the requirement for MV and CRRT.

### Statistical analysis

Statistical analysis was performed using IBM SPSS Statistics for Windows, Version 26 (IBM Corp, Armonk, NY, USA) and R for Windows, Version 4.3.0. The data were stratified into groups based on the presence or absence of malignancies among children with septic shock, denoted as the No Without and With groups, respectively. Continuous variables were presented as medians (interquartile range), while categorical variables were expressed as counts (frequency or percentage). The comparison of continuous variables utilized the Mann-Whitney U test, whereas the Chi-squared or Fisher's exact test was employed for the analysis of categorical variables. Kaplan-Meier survival curves, subjected to the log-rank test, were generated based on septic shock children with or without malignancies. Statistical significance was defined as p values < 0.05.

## Results

During the study period, a total of 508 children diagnosed with septic shock were enrolled (Table 1), comprising 85 patients with malignancies and 423 without malignancies. In the With group, 65 cases (76.5 %) were leukemia, and 20 cases (23.5 %) were solid tumors. The clinical characteristics of the children are summarized in Table 1, revealing that the With group exhibited a significantly older age (111 vs. 20 months, *p* < 0.001), lower levels of WBC (0.58 vs. 6.80 × 10^9/L, *p* < 0.001), Hb (80 vs. 98 × g/L, *p* < 0.001) and PLT (23 vs. 140 × 10^9/L, *p* < 0.001). Conversely, the With group had higher levels of bilirubin (11.50 vs. 6.60 μmol/L, *p* < 0.001). In terms of coagulation-related laboratory tests, the With group exhibited a shorter PT (15.7 vs. 17.1 s, *p* = 0.029) and APTT (43.2 vs. 47.3 s, *p* = 0.027). Moreover, the with group had higher levels of fibrinogen (2.61 vs. 2.37 g/L, *p* = 0.024) and lower levels of D-dimer (4.09 vs. 4.60 mg/L, *p* = 0.026) compared to the Without group. Notably, there were no significant differences in the use of vasoactive drugs (77.1 % vs. 80.0 %, *p* = 0.554), pSOFA score (9 vs. 10, *p* = 0.116) and PIM-3 score (0.07 vs. 0.06, *p* = 0.136). Regarding culture results, the With group exhibited a higher frequency of gram-negative organisms (37.6 % vs. 25.3 %, *p* = 0.028) and fungal organisms (15.3 % vs. 5.2 %, *p* = 0.002), whereas the Without group had a higher frequency of gram-positive organisms (21.0 % vs. 9.4 %, *p* = 0.019). Further analysis revealed that the requirements for CRRT were similar between the two groups (25.3 % vs. 28.2 %, *p* = 0.668), while MV showed a statistically significant difference (78.3 % vs. 57.6 %, *p* < 0.001). It is also noteworthy that the duration of CRRT days in the With group was significantly longer than that in the Without group (3 vs. 2 days, *p* = 0.019), while the duration of MV days was similar between the two groups (5 vs. 4 days, *p* = 0.458), as illustrated in Table 1.

**Table 1:** Demographics, clinical characteristics of children with or without malignancies.

Characteristics	Without (*N* = 423)	With (*N* = 85)	*p*
pSOFA, M (IQR)	9 (6-12)	10 (7-13)	0.116
PIM-3, M (IQR)	0.07 (0.04-0.12)	0.06 (0.03-0.12)	0.136
Age (month), M (IQR)	20 (6-84)	111 (60-153)	<0.001
Gender (male), *n* (%)	235 (55.6)	46 (54.1)	0.901
Culture results, *n* (%)			
Gram-positive	87 (20.6)	6 (7.1)	0.003
Gram-negative	116 (27.4)	37 (43.5)	0.003
Fungus	22 (5.2)	13 (15.3)	0.001
WBC (x10^9^ /l), M (IQR)	6.80 (3.96-14.87)	0.58 (0.20-3.82)	<0.001
Hb (g/l), M (IQR)	98 (83-115)	80 (66-94)	<0.001
PLT (x10^9^ /l), M (IQR)	140 (66-275)	23 (10-49)	<0.001
PCT (ng/ml), M (IQR)	25.40 (3.48-82.83)	28.00 (2.29-100.00)	0.757
CRP (mg/l), M (IQR)	44 (9-81)	42 (11-95)	0.866
Lactate (mmol/L), M (IQR)	2.00 (1.10-4.45)	1.80 (1.00-5.20)	0.726
Creatinine (mg/dL), M (IQR)	0.60 (0.30-1.00)	0.50 (0.30-1.10)	0.982
Bilirubin (μmol/L), M (IQR)	6.60 (3.15-15.80)	11.50 (6.20-24.40)	<0.001
PT(s), M (IQR)	17.1 (14.1-22.4)	15.7 (14.1-18.2)	0.029
INR, M (IQR)	1.43 (1.20-1.88)	1.36 (1.18-1.55)	0.054
APTT(s), M (IQR)	47.3 (35.9-68.6)	43.2 (35.5-51.1)	0.027
Fibrinogen (g/l), M (IQR)	2.37 (1.25-3.90)	2.61 (1.94-4.33)	0.024
D-dimer(mg/l), M (IQR)	4.60 (2.15-11.21)	4.09 (1.69-6.30)	0.026
Use of vasoactive drugs within 24 h, *n* (%)	326 (77.1)	68 (80.0)	0.554
Need of MV, *n* (%)	331 (78.3)	49 (57.6)	<0.001
The length of MV (days), M (IQR)	4 (1-9)	5 (1-8)	0.458
Need of CRRT, *n* (%)	107 (25.3)	24 (28.2)	0.668
The length of CRRT (days), M (IQR)	2 (1-4)	3 (2-4)	0.019

APTT, activated partial thromboplastin time; CRRT, continuous renal replacement therapy; INR, international normalized ratio; IQR, interquartile range; M, median; MV, mechanical ventilation; PLT, platelet; PCT, procalcitonin; s, second; pSOFA, pediatric sequential organ failure assessment; WBC, white blood cells.

The percentage composition of patients with acute kidney injury (AKI) (28.1 % vs. 22.4 %), gastrointestinal bleeding (7.8 % vs. 12.9 %), acute respiratory distress syndrome (ARDS) (7.3 % vs. 4.7 %), immunodeficiency (5.2 % vs. 1.0 %), and rheumatism (8.7 % vs. 3.5 %) did not exhibit significant differences between the two groups (Supplemental Table 1). However, concerning the composition of patients with respiratory failure (76.8 % vs. 64.7 %, *p* = 0.027), the Without group showed a significantly higher proportion than the With group.

The With group are more likely to have blood system infections (24.7 % vs. 3.3 %, *p* < 0.001), while the other group is more likely to have digestive system (33.7 % vs. 22.3 %, *p* = 0.009) and nervous system (9.6 % vs. 1.2 %, *p* = 0.010) infections (Table 2). A small proportion (7.8 %) of children in the without group have skin and tissue infections. There was no statistically significant difference between the two groups of children in terms of respiratory system infections (35.6 % vs. 27.2 %, *p* = 0.126).

**Table 2:** Primary infection sites.

Infection sites	Without (*N* = 423)	With (*N* = 85)	*p*
digest system, *n* (%)	157 (37.1)	19 (22.3)	0.009
respiratory system, *n* (%)	151 (35.6)	23 (27.0)	0.126
blood system, *n* (%)	14 (3.3)	21 (24.7)	<0.001
skin and soft tissue, *n* (%)	33 (7.8)	-	-
nervous system, *n* (%)	41 (9.6)	1 (1.2)	0.010
urinary system, *n* (%)	2 (0.4)	1 (1.2)	0.440
unknown, *n* (%)	25 (5.9)	19 (23.5)	<0.001

Regarding outcome indicators (Table 3), there were no statistically significant differences in the length of stay (LOS) in the PICU (5 vs. 5 days, *p* = 0.591), in-hospital mortality (43.0 % vs. 49.4 %, *p* = 0.276), and 28-day mortality (49.2 % vs. 44.7 %, *p* = 0.452). However, the LOS of the hospital (11 vs. 21 days, *p* < 0.001) in the Without group was shorter compared to the With group. The 28-day survival analysis (*p* = 0.314) also showed no significant differences.

**Table 3:** Clinical outcomes.

Characteristics	Without (*N* = 423)	With (*N* = 85)	*p*
28-day mortality, *n* (%)	208 (49.2)	38 (44.7)	0.452
In-hospital mortality, *n* (%)	182 (43.0)	42 (49.4)	0.276
LOS of PICU (days), M (IQR)	5 (2-10)	5 (2-8)	0.591
LOS of hospital (days),M (IQR)	11 (3-22)	21 (9-35)	<0.001

IQR, interquartile range; LOS, length of stay; M, median; PICU, pediatric intensive care unit.

Supplemental Table 2 shows the differences in etiology between the two groups. The Fungus (15.3 % vs. 4.5 %, p<0.001), Klebsiella (15.3 % vs. 4.0 %, p<0.001), and Escherichia coli (9.4 % vs. 3.8 %, *p* = 0.026) infected in the With group were significantly higher than those in the Without group, while streptococcus (1.1 % vs. 7.1 %, *p* = 0.038) were significantly lower than those in the without group.

**Fig. 1:**
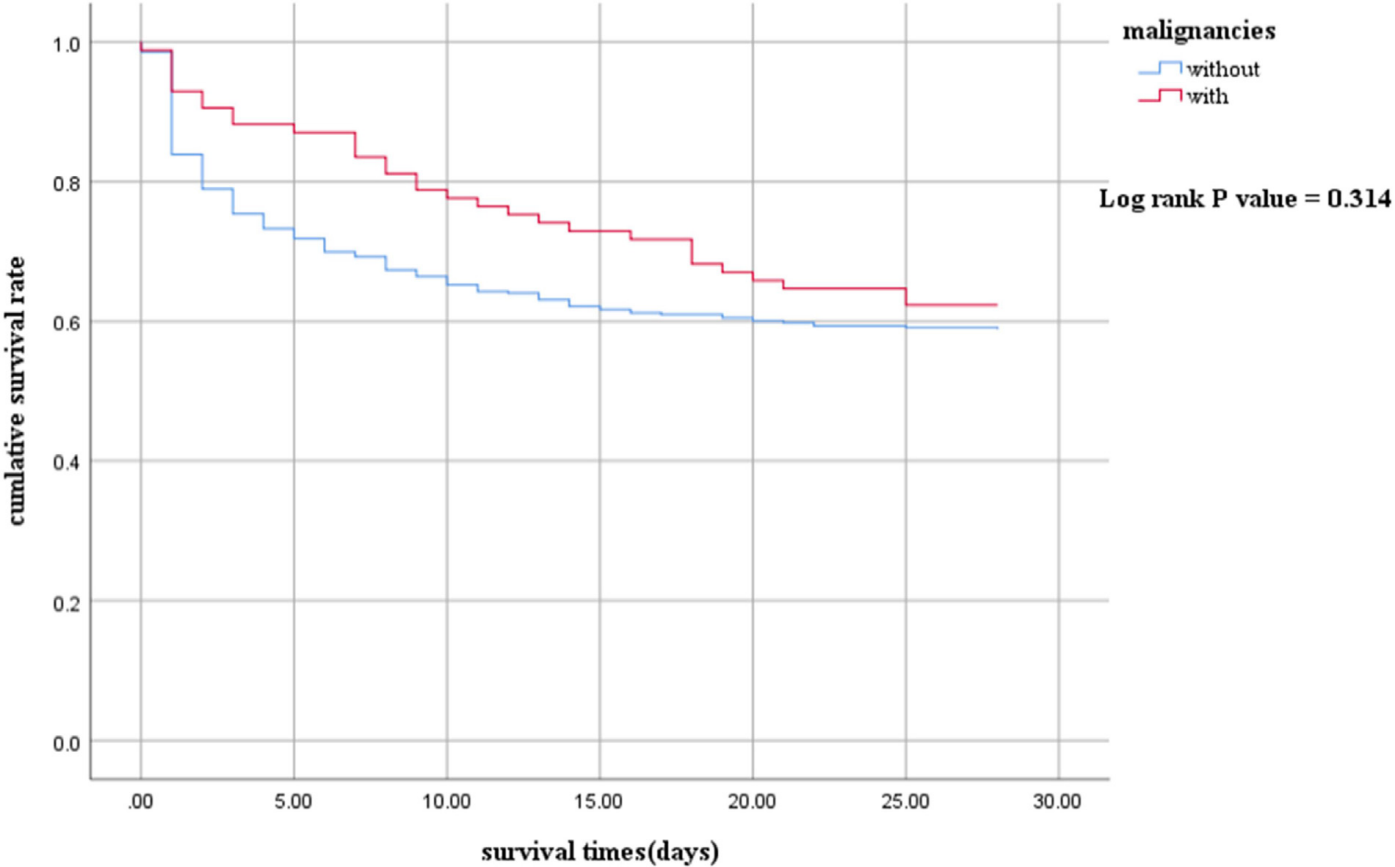
Kaplan–Meier survival curves between With and Without groups. The survival curves censored to 28-days.

The 28-day survival rate of the with group was higher than that of the without group, but there was no statistical significance. (log-rank *p* = 0.314) (Figure 1 Kaplan–Meier survival curves between With and Without groups. The survival curves censored to 28 days.).

## Discussion

Septic shock in pediatric patients with malignancies has consistently drawn the focus of the PICU. In the present study, the authors conducted a retrospective analysis of children experiencing septic shock who were admitted to the PICU within a pediatric hospital over the preceding eight years. The discernment revealed that (1) Septic shock children with malignancies have a longer hospital stays compared to those without malignancies. (2) The mortality rate of septic shock children with malignancies is not significantly different from those without malignancies. (3) The types of pathogens infected are significantly different between the two groups. (4) There are remarkable differences in the site of infection. (5) The incidence of MV support rates is different between the two groups.

Children with hematological malignancies and solid tumors usually require chemotherapy, which can cause organ dysfunction, myelosuppression, immune suppression, and susceptibility to various serious infections. This type of child is a high-risk group for sepsis and septic shock. The 28-day mortality rate and hospitalization mortality rate of septic shock children with malignancies in this study were 44.7 % and 49.4 %, respectively. A single-center study^13^ a decade ago reported a mortality rate of 52 % in ICU for sepsis children with leukemia. A recent review^14^ illuminated that the mortality rate of PICU in pediatric cancer patients with sepsis was 46.2 % (95 % CI, 34.7-57.8), which is similar to the mortality rate in the present study.

Intriguingly, this study first demonstrated that notwithstanding those with malignancies exhibited inferior indicators and longer hospital stays, the similarities in mortality rates between children with malignancies and those without malignancies in septic shock. This phenomenon could be attributed to improvements in sepsis management, advances in the treatment of malignancies, and enhancements in admission policies and technologies within the realm of the PICU. There is no statistically significant difference in the duration of anti-shock treatment between the two groups in the PICU. However, children with malignancies have longer hospital stays, which may be related to the treatment plan for malignancies. Notably, one study^15^ pointed out similar durations of hospitalization. A systematic review and meta-analysis indicated that the mortality rate of pediatric cancer patients in PICU was much higher than that of the general PICU population.^14^ However, their study did not compare the impact of septic shock on the prognosis of children with malignancies and non-malignancies.^14^

With the development of science and medicine, the success rate of treatment for hematological malignancies and solid tumors is increasing. Physicians, families, and patients are vigilant about the signs of infection in patients with malignant diseases and take proactive measures to diagnose and treat sepsis. Cuenca et al.^8^ showed that sepsis-related mortality rates were decreasing in cancer patients, while there was no significant change in non-cancer patients. This study showed no statistically significant difference in mortality rates caused by septic shock between children with malignancies and those without malignancies. Configuring better medical resources to treat children with malignancies who suffer from severe infections may be an effective measure to further improve outcomes.

In this study, the incidence of Gram-negative bacterial infections, notably Klebsiella and Escherichia coli, demonstrated an elevated occurrence in septic shock children with malignancies. Fungal infections, predominantly Candida, secured the second position, while Gram-positive bacterial involvement primarily implicated Staphylococcus. Recent research studies have corroborated similar observations, highlighting the prevalence of Gram-negative bacteria, including Klebsiella and Escherichia coli, in pediatric septic shock with malignancies. A study^16^ on pediatric cancer in blood cultures mentioned Gram-positive (15.0 %), Gram-negative (24.1 %), and Fungal (7.1 %), with Pseudomonas (3.4 %), Acinetobacter (3.0 %), Klebsiella (3.0 %), Escherichia coli (2.6 %), and Candida (6.8 %) being the main ones. In another study,^15^ the leading six pathogens were Klebsiella pneumoniae (7.9 %), Coagulase-negative Staphylococcus (5.8 %), Escherichia coli (5.8 %), Pseudomonas aeruginosa (5.0 %), Staphylococcus aureus (4.3 %), and Candida (4.3 %). Trehan et al.^17^ conducted a study on invasive bacterial infections in pediatric tumors, and the pathogens were mainly distributed in Escherichia coli (19 %), CoNS (Staphylococcus epidermidis, Staphylococcus hominis and Staphylococcus haemolyticus) (17 %), Staphylococcus aureus (15 %), Klebseilla pneumoniae (15 %), Pseudomonas aeruginosa (7 %).

Fungal infection is also an important issue that cannot be ignored in malignant disease. One study even mentioned that the fungal infection rate in the sepsis death group of children with tumors reached 17.7 %.^18^ In this study, the fungal infection rate in children with malignancies was as high as 15.3 %. In addition to conducting pathogen screening, when children with malignant diseases develop septic shock, empirical anti-infection plans must carefully consider the issues of Gram-negative bacterial and fungal infections.

Screening and judgment of the site of infection are also crucial for anti-infection treatment. Trehan et al.^17^ also noted that blood infection is much higher than other infection sites (Blood 99, Line 7, Pus 8, Urine 5), a phenomenon echoed in another study.^15^ In this study, blood infection in children with malignancies was also the primary site of infection (24.7 %), and a considerable number of patients (23.5 %) were difficult to determine the initial site of infection due to rapid disease progression, negative-culture, or blood culture combined with positive-culture from other parts. Both the high rates of fungal infection and blood infection may be linked to bone marrow suppression in children with malignancies after chemotherapy, rendering them more susceptible to infections and less capable of resisting fungal invasion. The lower levels of WBC, Hb and PLT support this inference. The fungal infection rate and blood infection rate were significantly lower in children without malignancies.

Conversely, septic shock children without malignancies exhibited an increased need for MV, potentially correlated with a higher incidence of respiratory failure and elevated rates of gastrointestinal and respiratory infections within this cohort. Nevertheless, both the demand for MV and CRRT in this study were inconsistent with the rates documented in certain other studies about children with malignancies. A study^19^ mentioned that the rate of mechanical ventilation was 66 % in children with hematological malignancies admitted to PICU due to respiratory failure. Singer et al.^13^ conducted a study on acute leukemia sepsis in pediatric intensive care units, revealing a demand for MV of 58 % and CRRT of 39 %. Another study^20^ about children with cancer in a PICU reported a usage rate of only 3.9 % for RRT. This difference may be related to the differences in the included population, as this study focused on children with critically severe conditions such as septic shock. As of now, the authors have not encountered recent research suitable for a comprehensive comparison. Future investigations with larger sample sizes are imperative to assess the impact of MV and CRRT on mortality in children experiencing septic shock with malignancies.

Moreover, this study indicated that children with malignancies are of advanced age, which may be linked to the onset age of the disease. Several studies^13^^,^^15^ on childhood tumors are concentrated around the age of 10 years (Singer et al: 12-14 years, Rafael T: 99 months), supporting the findings of this study.

### Limitations

There are several limitations to this study. First, these findings are based on data from a single center, which may pose a risk of bias. The admission criteria for PICU in different hospitals may vary, so this study selected patients with septic shock to ensure better comparability in different centers. Second, the sample size is relatively small. Third, the staging/grading of some patients was not completely clear due to the death after admission, so some confounding factors cannot be ruled out. Fourth, there was a significant age difference between children with malignancies and those without malignancies. Fifth, the treatment of the two groups of children is not completely consistent. Looking forward to future multicenter large-scale studies exploring the prognosis of septic shock in children with malignancies.

## Conclusion

Although the bacterial spectrum and site of infection were different, the septic shock children with malignancies versus those without malignancies showed a similar mortality rate. The septic shock children with malignancies had long LOS of a hospital. Pediatricians should properly arrange medical resources and actively treat children with malignant diseases that develop septic shock.

## Conflicts of interest

The authors declare no conflicts of interest.

## Appendix Supplementary materials

Image, application 1
