# Supplementary material for: Comparing the clinical characteristics and outcomes of septic shock children with and without malignancies: a retrospective cohort study
Source: J Pediatr (Rio J). 2024 Jul 3;100(6):633–9. doi: 10.1016/j.jped.2024.06.003 (PMC11662747; doi:10.1016/j.jped.2024.06.003)
Supplement: Supplementary file 1 [file mmc1.docx]

JPED-D-24-00118_Supplementary Material

Supplemental Table 1. Organ dysfunction and Basic diseases

|  | Without（N=423） | With （N=85） | P |
| --- | --- | --- | --- |
| Respiratory failure, n （%) | 325(76.8) | 55(64.7) | 0.027 |
| AKI, n （%) | 119(28.1) | 19(22.4) | 0.337 |
| Gastrointestinal bleeding, n （%) | 33(7.8) | 11(12.9) | 0.185 |
| ARDS, n （%) | 31(7.3) | 4(4.7) | 0.524 |

AKI, acute kidney injury; ARDS, acute respiratory distress syndrome; DIC, disseminated intravascular coagulation.

Supplemental Table 2 Frequency of bacteria for the positive cultures

| Species | Without（N=423） | With （N=85） | p |
| --- | --- | --- | --- |
| Gram-positive | 87(20.6) | 6(7.1) | 0.003 |
| Staphylococcus species | 47(11.1) | 5(5.9) | 0.147 |
| Streptococcus species | 30(7.1) | 1(1.1) | 0.038 |
| Others | 10(2.4) | - |  |
| Gram-negative | 116(27.4) | 37(43.5) | 0.003 |
| Klebsiella species | 17(4.0) | 13(15.3) | ＜0.001 |
| Pseudomonas aeruginosa | 23(5.4) | 5(5.9) | 0.870 |
| Escherichia coli | 16(3.8) | 8(9.4) | 0.026 |
| Acinetobacter baumannii | 19(4.5) | 3(3.5) | 0.691 |
| Salmonella | 5(1.2) | 3(3.5) | 0.113 |
| Others | 36(8.5) | 5(5.9) | 0.417 |
| Fungus | 22(5.2) | 13(15.3) | 0.001 |
| Candida | 19(4.5) | 13(15.3) | ＜0.001 |
| Cryptococcus neoformans | 2(0.5) | - |  |
| Cyanobacteria marneffei， | 1(0.2) | - |  |

Others included：Aeromonas hydrophila, Burkholderia cepacia, Enterobacter cloacae, Feces Enterococcus，Haemophilus influenzae, Moraxella catarrhalis, Neisseria meningitides, Ralstonia mannitollytica, Stenotrophomonas maltophilia.
